# Crystal structure and Hirshfeld surface analysis of (2*E*)-3-(3-bromo-4-fluoro­phen­yl)-1-(3,4-di­meth­oxy­phen­yl)prop-2-en-1-one

**DOI:** 10.1107/S2056989018009416

**Published:** 2018-07-06

**Authors:** S. N. Sheshadri, Zeliha Atioğlu, Mehmet Akkurt, M. K. Veeraiah, Ching Kheng Quah, C. S. Chidan Kumar, B. P. Siddaraju

**Affiliations:** a Department of Chemistry, GSSS Institute of Engineering & Technology for Women, Mysuru 570 016, Karnataka, India; b İlke Education and Health Foundation, Cappadocia University, Cappadocia Vocational College, The Medical Imaging Techniques Program, 50420 Mustafapaşa, Ürgüp, Nevşehir, Turkey; c Department of Physics, Faculty of Sciences, Erciyes University, 38039 Kayseri, Turkey; d Department of Chemistry, Sri Siddhartha Institute of Technology, Tumkur 572 105, Karnataka, India; e X-ray Crystallography Unit, School of Physics, Universiti Sains Malaysia, 11800 USM, Penang, Malaysia; f Department of Engineering Chemistry, Vidya Vikas Institute of Engineering & Technology, Visvesvaraya Technological University, Alanahalli, Mysuru 570 028, Karnataka, India; g Department of Chemistry, Cauvery Institute of Technology, Mandya 571 402, Karnataka, India

**Keywords:** crystal structure, hydrogen contacts, 3-bromo-4-fluoro­phenyl ring, di­meth­oxy­phenyl ring, Hirshfeld surface

## Abstract

In the mol­ecule of the title compound, C_17_H_14_BrFO_3_, the aromatic rings are tilted with respect to the enone bridge by 13.63 (14) and 4.27 (15)°, and form a dihedral angle 17.91 (17)°. In the crystal, centrosymmetrically related mol­ecules are linked by pairs of C—H⋯O hydrogen bonds into dimeric units, forming rings of *R*_2_^2^(14) graph-set motif. The dimers are further connected by weak C—H⋯O hydrogen inter­actions, forming layers parallel to (10). Hirshfeld surface analysis shows that van der Waals inter­actions constitute the major contribution to the inter­molecular inter­actions, with H⋯H contacts accounting for 29.7% of the surface.

## Abstract 2

The title compound is constructed from two aromatic rings (3-bromo-4-fluoro­phenyl and 3,4-di­meth­oxy­phen­yl), which are linked by a C=C—C(=O)—C enone bridge and form a dihedral angle of 17.91 (17)°. In the crystal, mol­ecules are linked by C—H⋯O hydrogen bonds enclosing rings of 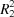
(14) graph-set motif to form layers parallel to (10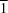).

## Chemical context   

Natural products are important sources to search for new agents for cancer therapies with minimal side effects. Chalcones, which are considered to be the precursors of flavonoids and isoflavonoids, are abundant in edible plants. They consist of open-chain flavonoids in which the two aromatic rings are joined by a three-carbon α,β-unsaturated carbonyl system. These are coloured compounds because of the presence of the –CO—CH=CH– chromophore, the colour depending on the presence of other auxochromes. Accumulating evidence has shown that chalcones and their derivatives could inhibit tumor initiation and progression. In view of the above and in a continuation of our previous work on 3,4-dimeth­oxy chalcones (Sheshadri *et al.*, 2018 ▸), herewith we report the crystal and mol­ecular structures of the title compound.
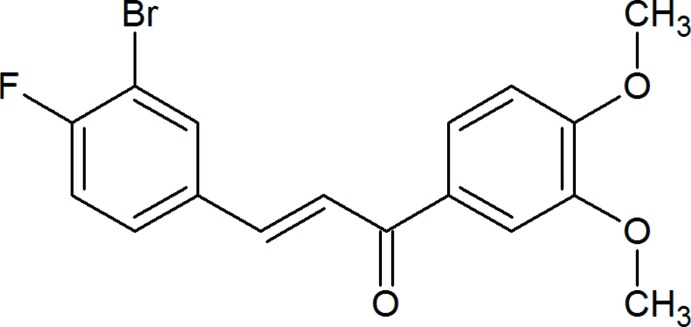


## Structural commentary   

**Figure 1:**
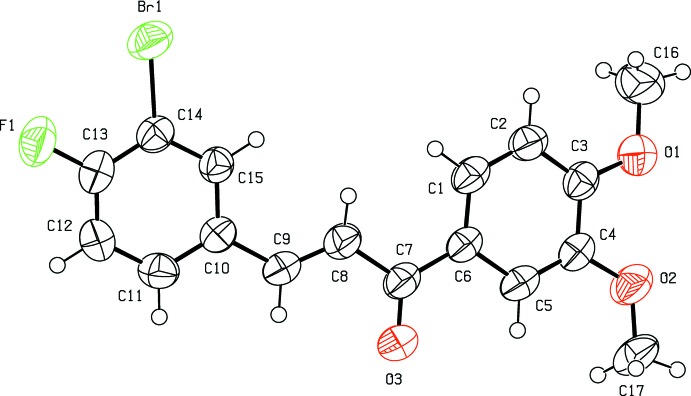
The mol­ecular structure of the title compound with displacement ellipsoids drawn at the 50% probability level.

The title compound (Fig. 1 ▸) is constructed by two aromatic rings (3-bromo-4-fluoro­phenyl and a 3,4-di­meth­oxy­phen­yl), which are linked by a C=C—C(=O)—C enone bridge. The mol­ecule is twisted substanti­ally about the enone bridge, as indicated by the dihedral angles of 13.63 (14) and 4.27 (15)° formed by the mean plane through C7–C8/O3 [maximum deviation 0.045 (4) Å for atom C7] and the C1–C6 and C10–C15 aromatic rings. The dihedral angle between the mean planes of the 3,4- meth­oxy­phenyl and 3-bromo-4-fluoro­phenyl rings is 17.91 (17)°. The H atoms of the central propenone group are *trans* configured. The two meth­oxy groups attached to C16 and C17 are almost coplanar with the benzene ring, with the deviations of 0.333 (6) Å for C16 and −0.124 (4) Å for C17. The bond lengths and angles are comparable with those found in the related compounds (2*E*)-3-(3-chloro­phen­yl)-1-(3,4-di­meth­oxy­phen­yl)-prop-2-en-1-one (Sheshadri *et al.*, 2018 ▸), (*E*)-3-(3,4- di­meth­oxy­phen­yl)-1-(1-hy­droxy­naph­th­al­en-2­yl)prop-2-en-1-one (Ezhilarasi *et al.*, 2015 ▸), (*E*)-1-(3-bromo­phen­yl)-3-(3,4-di­meth­oxy­phen­yl)prop-2-en-1-one (Esc­o­bar *et al.*, 2012 ▸) and (*E*)-3-(2-bromo­phen­yl)-1-(3,4-di­meth­oxy­phen­yl)prop-2-en-1-one (Li *et al.*, 2012 ▸).

## Supra­molecular features   

**Figure 2:**
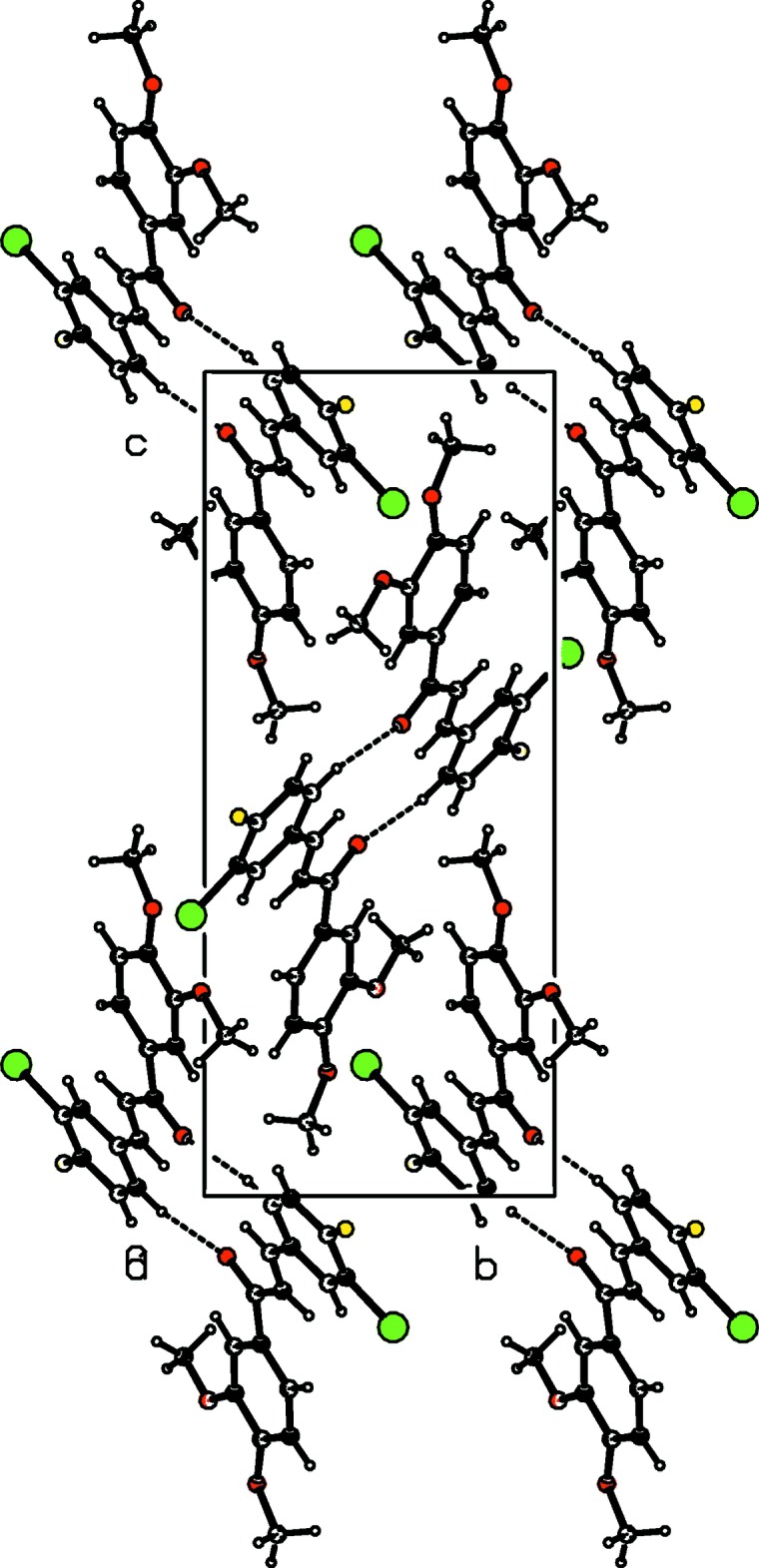
A view along the *a* axis of the crystal packing of the title compound. H atoms not involved in hydrogen bonding (dashed lines) are omitted for clarity.

In the crystal, centrosymmetrically related mol­ecules are linked by pairs of C—H⋯O hydrogen bonds into dimers forming rings with an 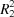 (14) graph-set motif (Table 1 ▸, Fig. 2 ▸). The dimeric units are further connected by weak C—H⋯O hydrogen bonds, forming layers parallel to (10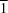).

In addition, weak C—Br⋯π [C14—Br1 = 1.877 (3) Å, Br1⋯*Cg*1^i^ = 3.7959 (16) Å, C14⋯*Cg*1^i^ = 4.010 (4) Å, C14—Br1⋯*Cg*1^i^ = 82.54 (11)°; symmetry code: (i) −1 + *x*, *y*, *z*; *Cg*1 is the centroid of the C1–C6 ring] and C—F⋯π [C13—F1 = 1.348 (4) Å, F1⋯*Cg*2^ii^ = 3.454 (3) Å, C13⋯*Cg*2^ii^ = 3.659 (4) Å, C13—F1⋯*Cg*2^ii^ = 87.78 (19)°; symmetry code: (ii) −*x*, 1 − *y*, −*z*; *Cg*2 is the centroid of the C10–C15 ring] inter­actions help to stabilize the crystal structure.

## Hirshfeld Surface Analysis   

**Figure 3:**
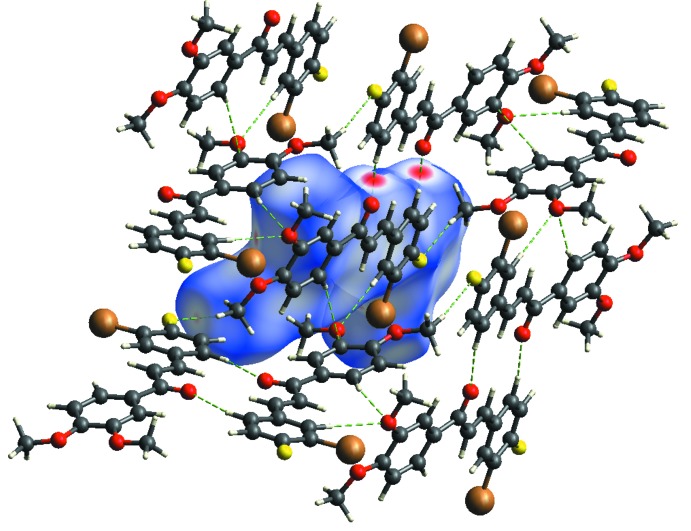
The Hirshfeld surface mapped over *d*_norm_ showing the C—H⋯O and C—H⋯F contacts.

**Figure 4:**
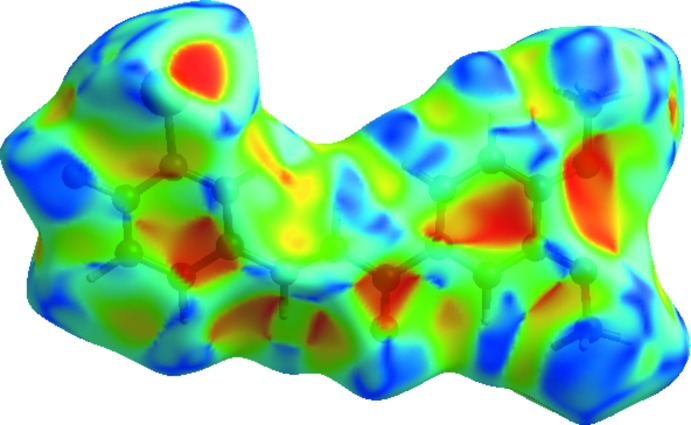
Hirshfeld surface of the title compound plotted over shape-index.

**Figure 5:**
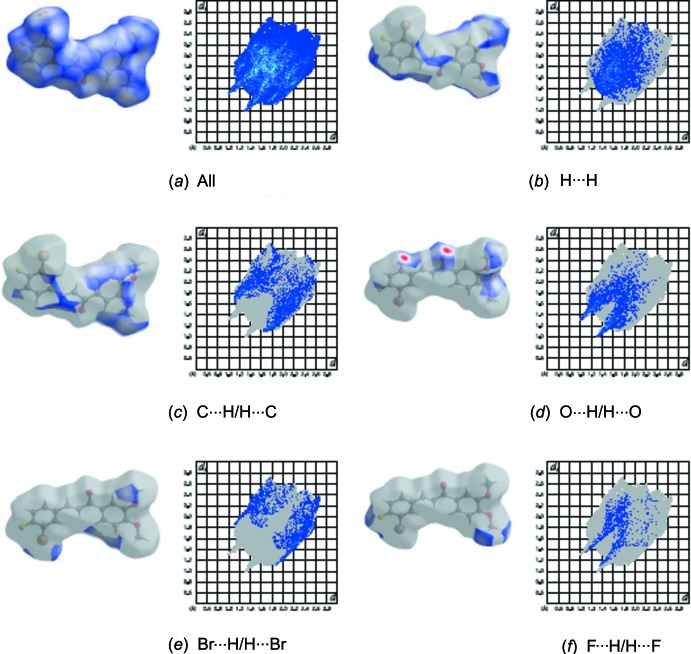
The two-dimensional fingerprint plots of the title compound, showing (*a*) all inter­actions, and delineated into (*b*) H⋯H, (*c*) C⋯H/H⋯C, (*d*) O⋯H/H⋯O, (*e*) Br⋯H/H⋯Br and (*f*) F⋯H/H⋯F inter­actions [*d*_e_ and *d*_i_ represent the distances from a point on the Hirshfeld surface to the nearest atoms outside (external) and inside (inter­nal) the surface, respectively].

Mol­ecular Hirshfeld surfaces (Hirshfeld, 1977 ▸; Spackman & Jayatilaka, 2009 ▸) are constructed based on the electron distribution calculated as the sum of spherical atom electron densities (Spackman & Byrom, 1997 ▸). Hirshfeld surface analysis is a tool for visualizing the inter­molecular inter­actions; it can include comparisons to the van der Waals envelope, which other mol­ecules or atoms come into contact with when inter­actions are present. The Hirshfeld surface and two-diensional fingerprint plots of the title compound were calculated using *CrystalExplorer17.5* (Turner *et al.*, 2017 ▸). In the Hirshfeld surface plotted over *d*_norm_ (Fig. 3 ▸), the white surfaces indicate contacts with distances equal to the sum of van der Waals radii, and the red and blue colours indicate distances shorter or longer than the van der Waals radii, respectively (Venkatesan *et al.*, 2016 ▸). The bright-red spots appearing near to O2, F1, Br1 and hydrogen atoms H15*A*, H16*A*, H17*C* indicate their role as donors and acceptors in the dominant C—H⋯O, C—H⋯F and C—H⋯Br contacts. The shape-index of the Hirshfeld surface is a tool to visualize the π–π stacking inter­actions by the presence of adjacent red and blue triangles; if there are no adjacent red and/or blue triangles, then there are no π–π inter­actions. The Hirshfeld surface of the title compound plotted over shape-index (Fig. 4 ▸) clearly suggest that this is the case here. The overall two-dimensional fingerprint plot and those delineated into H⋯H, C⋯H/H⋯C, O⋯H/H⋯O, Br⋯H/H⋯Br and F⋯H/H⋯F contacts (McKinnon *et al.*, 2007 ▸) are illustrated in Fig. 5 ▸ *a*–*f*, respectively. Their relative contributions to the Hirshfeld surface are given in Table 2 ▸. The most important inter­action is H⋯H, contributing 29.7% to the overall crystal packing, which is reflected as widely scattered points of high density due to the large hydrogen content of the mol­ecule. In the absence of C—H⋯π inter­actions in the crystal, shown as a pair of characteristic wings the fingerprint plot, H⋯C/C⋯H contacts contribute 19.2% to the Hirshfeld surface (Fig. 5 ▸ *c*). The O⋯H/H⋯O, Br⋯C/C⋯Br and F⋯C/C⋯F contacts in the structure with 17.9, 5.6 and 5.0% contributions, respectively, to the Hirshfeld surface have a symmetrical distribution of points (Fig. 5 ▸ *d*–*f*). The other Br⋯C / C⋯Br, F⋯C / C⋯F, C⋯C, F⋯O / O⋯F and C⋯O / O⋯C contacts, having only small contributions to the Hirshfeld surface, have negligible directional impact on the mol­ecular packing.

## Synthesis and crystallization   

The reagents and solvents for the synthesis were obtained from the Aldrich Chemical Co., and were used without additional purification. The title compound was synthesized as per the procedure reported earlier (Kumar *et al.*, 2013*a* ▸,*b* ▸; Chidan Kumar *et al.*, 2014 ▸). 1-(3,4-Di­meth­oxy­phen­yl) ethanone (0.01mol) and 3-bromo-4-fluoro­benzaldehyde (0.01mol) were dissolved in 20 ml methanol. A catalytic amount of NaOH was added to the solution dropwise with vigorous stirring. The reaction mixture was stirred for about 6 h at room temperature. The progress of the reaction was monitored by TLC. The formed crude product was filtered, washed repeatedly with distilled water and recrystallized from ethanol to obtain the title chalcone. Crystals suitable for X-ray diffraction studies were obtained from an acetone solution by the slow evaporation technique at room temperature. The melting point (381–383 K) was determined by a Stuart Scientific (UK) apparatus. The purity of the compound was confirmed by thin layer chromatography using Merck silica gel 60 F254 coated aluminum plates.

## Refinement   

Crystal data, data collection and structure refinement details are summarized in Table 3 ▸. C-bound H atoms were positioned geometrically and refined using a riding model, with C—H = 0.93–0.6 Å, and with *U*_iso_(H) = 1.2*U*_eq_(C) or 1.5*U*_eq_(C) for methyl H atoms.

## Supplementary Material

Crystal structure: contains datablock(s) I. DOI: 10.1107/S2056989018009416/rz5240sup1.cif

Structure factors: contains datablock(s) I. DOI: 10.1107/S2056989018009416/rz5240Isup2.hkl

CCDC reference: 1852842

Additional supporting information:  crystallographic information; 3D view; checkCIF report

## Figures and Tables

**Table 1:** Hydrogen-bond geometry (Å, °)

*D*—H⋯*A*	*D*—H	H⋯*A*	*D*⋯*A*	*D*—H⋯*A*
C15—H15*A*⋯O2^i^	0.93	2.61	3.506 (5)	162
C11—H11*A*⋯O3^ii^	0.93	2.46	3.358 (5)	162

Symmetry codes: (i) 
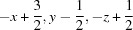
; (ii) 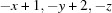.

**Table 2:** Percentage contributions of inter­atomic contacts to the Hirshfeld surface for the title compound

Contact	Percentage contribution
H⋯H	29.7
C⋯H/H⋯C	19.2
O⋯H/H⋯O	17.9
Br⋯H/H⋯Br	11.2
F⋯H/H⋯F	6.8
Br⋯C/C⋯Br	5.6
F⋯C/C⋯F	5.0
C⋯C	3.1
F⋯O/O⋯F	0.7
C⋯O/O⋯C	0.4

**Table 3:** Experimental details

Crystal data	
Chemical formula	C_17_H_14_BrFO_3_
*M*_r_	365.19
Crystal system, space group	Monoclinic, *P*2_1_/*n*
Temperature (K)	294
*a*, *b*, *c* (Å)	8.9212 (12), 8.6601 (11), 20.538 (3)
β (°)	96.896 (3)
*V* (Å^3^)	1575.2 (4)
*Z*	4
Radiation type	Mo *K*α
μ (mm^−1^)	2.63
Crystal size (mm)	0.31 × 0.30 × 0.11

Data collection	
Diffractometer	Bruker APEXII CCD
Absorption correction	Multi-scan (*SADABS*; Sheldrick, 2007 ▸)
*T*_min_, *T*_max_	0.465, 0.755
No. of measured, independent and observed [*I* > 2σ(*I*)] reflections	11919, 3240, 2287
*R*_int_	0.031
(sin θ/λ)_max_ (Å^−1^)	0.627

Refinement	
*R*[*F* ^2^ > 2σ(*F* ^2^)], *wR*(*F* ^2^), *S*	0.046, 0.149, 1.05
No. of reflections	3240
No. of parameters	199
H-atom treatment	H-atom parameters constrained
Δρ_max_, Δρ_min_ (e Å^−3^)	0.78, −0.66

Computer programs: *APEX2* and *SAINT* (Bruker, 2007 ▸), *SHELXS97* (Sheldrick, 2008 ▸), *SHELXL2014* (Sheldrick, 2015 ▸), *ORTEP-3 for Windows* (Farrugia, 2012 ▸) and *PLATON* (Spek, 2009 ▸).

## supplementary crystallographic information

### Crystal data

**Table:**

C_17_H_14_BrFO_3_	*F*(000) = 736
*M**_r_* = 365.19	*D*_x_ = 1.540 Mg m^−^^3^
Monoclinic, *P*2_1_/*n*	Mo *K*α radiation, λ = 0.71073 Å
*a* = 8.9212 (12) Å	Cell parameters from 3205 reflections
*b* = 8.6601 (11) Å	θ = 2.4–22.9°
*c* = 20.538 (3) Å	µ = 2.63 mm^−^^1^
β = 96.896 (3)°	*T* = 294 K
*V* = 1575.2 (4) Å^3^	Block, colourless
*Z* = 4	0.31 × 0.30 × 0.11 mm

### Data collection

**Table:**

Bruker APEXII CCD diffractometer	2287 reflections with *I* > 2σ(*I*)
φ and ω scans	*R*_int_ = 0.031
Absorption correction: multi-scan (SADABS; Sheldrick, 2007)	θ_max_ = 26.5°, θ_min_ = 2.4°
*T*_min_ = 0.465, *T*_max_ = 0.755	*h* = −11→10
11919 measured reflections	*k* = −10→10
3240 independent reflections	*l* = −25→25

### Refinement

**Table:**

Refinement on *F*^2^	0 restraints
Least-squares matrix: full	Hydrogen site location: inferred from neighbouring sites
*R*[*F*^2^ > 2σ(*F*^2^)] = 0.046	H-atom parameters constrained
*wR*(*F*^2^) = 0.149	*w* = 1/[σ^2^(*F*_o_^2^) + (0.0824*P*)^2^ + 0.5509*P*] where *P* = (*F*_o_^2^ + 2*F*_c_^2^)/3
*S* = 1.05	(Δ/σ)_max_ < 0.001
3240 reflections	Δρ_max_ = 0.78 e Å^−^^3^
199 parameters	Δρ_min_ = −0.65 e Å^−^^3^

### Special details

**Table:**

Geometry. All esds (except the esd in the dihedral angle between two l.s. planes) are estimated using the full covariance matrix. The cell esds are taken into account individually in the estimation of esds in distances, angles and torsion angles; correlations between esds in cell parameters are only used when they are defined by crystal symmetry. An approximate (isotropic) treatment of cell esds is used for estimating esds involving l.s. planes.

### Fractional atomic coordinates and isotropic or equivalent isotropic displacement parameters (Å^2^)

**Table:**

	*x*	*y*	*z*	*U*_iso_*/*U*_eq_	
Br1	−0.03000 (5)	0.46149 (5)	0.15921 (2)	0.0788 (2)	
F1	−0.2029 (2)	0.5975 (3)	0.03979 (14)	0.0862 (7)	
O1	1.0604 (3)	0.8509 (4)	0.34912 (13)	0.0743 (8)	
O2	1.1326 (3)	0.9899 (4)	0.24774 (15)	0.0803 (8)	
O3	0.6831 (3)	0.9371 (4)	0.07316 (15)	0.0788 (8)	
C1	0.7272 (4)	0.7591 (4)	0.23364 (19)	0.0575 (9)	
H1A	0.636474	0.705562	0.231962	0.069*	
C2	0.8261 (4)	0.7559 (4)	0.29089 (18)	0.0589 (9)	
H2A	0.802350	0.699096	0.326693	0.071*	
C3	0.9606 (4)	0.8373 (4)	0.29488 (18)	0.0550 (8)	
C4	0.9977 (4)	0.9146 (4)	0.23886 (18)	0.0532 (8)	
C5	0.8997 (4)	0.9162 (4)	0.18250 (18)	0.0541 (8)	
H5A	0.925561	0.968527	0.145921	0.065*	
C6	0.7591 (3)	0.8392 (4)	0.17906 (17)	0.0498 (7)	
C7	0.6521 (4)	0.8551 (4)	0.11860 (18)	0.0570 (8)	
C8	0.5024 (4)	0.7777 (4)	0.11436 (19)	0.0606 (9)	
H8A	0.486595	0.702343	0.145064	0.073*	
C9	0.3917 (4)	0.8126 (4)	0.06853 (17)	0.0543 (8)	
H9A	0.414505	0.883278	0.037089	0.065*	
C10	0.2365 (4)	0.7534 (4)	0.06104 (17)	0.0517 (8)	
C11	0.1337 (4)	0.8057 (4)	0.01072 (18)	0.0620 (9)	
H11A	0.164224	0.877235	−0.018805	0.074*	
C12	−0.0149 (4)	0.7541 (5)	0.00294 (19)	0.0669 (10)	
H12A	−0.083256	0.790553	−0.031380	0.080*	
C13	−0.0592 (4)	0.6488 (4)	0.0466 (2)	0.0610 (9)	
C14	0.0408 (4)	0.5962 (4)	0.09817 (17)	0.0542 (8)	
C15	0.1889 (4)	0.6462 (4)	0.10559 (17)	0.0514 (8)	
H15A	0.256713	0.609080	0.139942	0.062*	
C16	1.0182 (6)	0.7928 (8)	0.4101 (2)	0.1109 (19)	
H16A	1.099315	0.809974	0.444494	0.166*	
H16B	0.929428	0.845664	0.420378	0.166*	
H16C	0.997848	0.684197	0.406049	0.166*	
C17	1.1885 (5)	1.0598 (5)	0.1947 (2)	0.0817 (13)	
H17A	1.283889	1.107434	0.209172	0.123*	
H17B	1.201710	0.983432	0.162030	0.123*	
H17C	1.118491	1.136934	0.176371	0.123*	

### Atomic displacement parameters (Å^2^)

**Table:**

	*U*^11^	*U*^22^	*U*^33^	*U*^12^	*U*^13^	*U*^23^
Br1	0.0718 (3)	0.0733 (3)	0.0950 (4)	−0.0108 (2)	0.0252 (2)	0.0155 (2)
F1	0.0526 (12)	0.0955 (17)	0.1080 (19)	−0.0115 (13)	−0.0013 (12)	−0.0013 (15)
O1	0.0636 (15)	0.090 (2)	0.0675 (17)	−0.0085 (15)	0.0020 (13)	0.0060 (14)
O2	0.0642 (17)	0.099 (2)	0.0778 (19)	−0.0323 (16)	0.0096 (14)	0.0020 (16)
O3	0.0607 (16)	0.095 (2)	0.0808 (19)	−0.0177 (15)	0.0098 (14)	0.0268 (16)
C1	0.0516 (19)	0.0472 (18)	0.076 (2)	−0.0054 (15)	0.0188 (17)	0.0007 (16)
C2	0.057 (2)	0.058 (2)	0.065 (2)	−0.0037 (17)	0.0155 (17)	0.0059 (16)
C3	0.0477 (17)	0.0525 (19)	0.066 (2)	0.0040 (15)	0.0128 (16)	−0.0029 (16)
C4	0.0463 (17)	0.0494 (17)	0.066 (2)	−0.0043 (15)	0.0138 (16)	−0.0054 (16)
C5	0.0530 (19)	0.0452 (17)	0.068 (2)	−0.0037 (15)	0.0226 (17)	−0.0006 (15)
C6	0.0457 (16)	0.0420 (16)	0.064 (2)	−0.0016 (14)	0.0159 (14)	−0.0011 (14)
C7	0.0489 (18)	0.0544 (19)	0.070 (2)	−0.0055 (16)	0.0150 (16)	0.0036 (17)
C8	0.052 (2)	0.059 (2)	0.071 (2)	−0.0067 (17)	0.0109 (17)	0.0097 (18)
C9	0.0520 (19)	0.054 (2)	0.059 (2)	−0.0062 (16)	0.0164 (16)	0.0026 (16)
C10	0.0502 (18)	0.0512 (19)	0.0548 (19)	−0.0021 (15)	0.0104 (15)	−0.0036 (14)
C11	0.069 (2)	0.058 (2)	0.059 (2)	−0.0038 (18)	0.0096 (17)	0.0078 (17)
C12	0.058 (2)	0.075 (2)	0.064 (2)	0.004 (2)	−0.0050 (17)	0.0049 (19)
C13	0.0466 (19)	0.061 (2)	0.075 (2)	−0.0020 (17)	0.0068 (17)	−0.0090 (18)
C14	0.0529 (19)	0.0468 (17)	0.064 (2)	−0.0014 (16)	0.0130 (16)	−0.0013 (15)
C15	0.0471 (17)	0.0505 (18)	0.057 (2)	0.0027 (15)	0.0079 (14)	0.0020 (15)
C16	0.104 (4)	0.155 (5)	0.069 (3)	−0.039 (4)	−0.006 (3)	0.020 (3)
C17	0.067 (3)	0.088 (3)	0.094 (3)	−0.030 (2)	0.025 (2)	0.000 (2)

### Geometric parameters (Å, º)

**Table:**

Br1—C14	1.877 (3)	C8—H8A	0.9300
F1—C13	1.348 (4)	C9—C10	1.466 (5)
O1—C3	1.345 (4)	C9—H9A	0.9300
O1—C16	1.441 (5)	C10—C11	1.374 (5)
O2—C4	1.362 (4)	C10—C15	1.405 (5)
O2—C17	1.390 (5)	C11—C12	1.390 (5)
O3—C7	1.231 (4)	C11—H11A	0.9300
C1—C6	1.377 (5)	C12—C13	1.371 (5)
C1—C2	1.383 (5)	C12—H12A	0.9300
C1—H1A	0.9300	C13—C14	1.377 (5)
C2—C3	1.385 (5)	C14—C15	1.381 (5)
C2—H2A	0.9300	C15—H15A	0.9300
C3—C4	1.405 (5)	C16—H16A	0.9600
C4—C5	1.364 (5)	C16—H16B	0.9600
C5—C6	1.414 (5)	C16—H16C	0.9600
C5—H5A	0.9300	C17—H17A	0.9600
C6—C7	1.479 (5)	C17—H17B	0.9600
C7—C8	1.487 (5)	C17—H17C	0.9600
C8—C9	1.315 (5)		
			
C3—O1—C16	118.2 (3)	C11—C10—C9	120.0 (3)
C4—O2—C17	119.9 (3)	C15—C10—C9	121.2 (3)
C6—C1—C2	122.0 (3)	C10—C11—C12	121.5 (3)
C6—C1—H1A	119.0	C10—C11—H11A	119.3
C2—C1—H1A	119.0	C12—C11—H11A	119.3
C1—C2—C3	120.0 (3)	C13—C12—C11	119.0 (3)
C1—C2—H2A	120.0	C13—C12—H12A	120.5
C3—C2—H2A	120.0	C11—C12—H12A	120.5
O1—C3—C2	125.2 (3)	F1—C13—C12	119.7 (3)
O1—C3—C4	116.0 (3)	F1—C13—C14	119.4 (3)
C2—C3—C4	118.8 (3)	C12—C13—C14	120.8 (3)
O2—C4—C5	125.1 (3)	C13—C14—C15	120.3 (3)
O2—C4—C3	114.2 (3)	C13—C14—Br1	118.7 (3)
C5—C4—C3	120.7 (3)	C15—C14—Br1	121.0 (3)
C4—C5—C6	120.8 (3)	C14—C15—C10	119.7 (3)
C4—C5—H5A	119.6	C14—C15—H15A	120.1
C6—C5—H5A	119.6	C10—C15—H15A	120.1
C1—C6—C5	117.7 (3)	O1—C16—H16A	109.5
C1—C6—C7	123.7 (3)	O1—C16—H16B	109.5
C5—C6—C7	118.5 (3)	H16A—C16—H16B	109.5
O3—C7—C6	120.6 (3)	O1—C16—H16C	109.5
O3—C7—C8	119.8 (3)	H16A—C16—H16C	109.5
C6—C7—C8	119.5 (3)	H16B—C16—H16C	109.5
C9—C8—C7	122.0 (3)	O2—C17—H17A	109.5
C9—C8—H8A	119.0	O2—C17—H17B	109.5
C7—C8—H8A	119.0	H17A—C17—H17B	109.5
C8—C9—C10	127.8 (3)	O2—C17—H17C	109.5
C8—C9—H9A	116.1	H17A—C17—H17C	109.5
C10—C9—H9A	116.1	H17B—C17—H17C	109.5
C11—C10—C15	118.7 (3)		
			
C6—C1—C2—C3	1.3 (5)	C5—C6—C7—C8	−178.8 (3)
C16—O1—C3—C2	−7.8 (6)	O3—C7—C8—C9	−11.1 (6)
C16—O1—C3—C4	171.3 (4)	C6—C7—C8—C9	165.6 (3)
C1—C2—C3—O1	175.4 (3)	C7—C8—C9—C10	−175.7 (3)
C1—C2—C3—C4	−3.7 (5)	C8—C9—C10—C11	178.4 (4)
C17—O2—C4—C5	−8.4 (6)	C8—C9—C10—C15	0.1 (6)
C17—O2—C4—C3	174.5 (4)	C15—C10—C11—C12	−0.4 (5)
O1—C3—C4—O2	1.4 (5)	C9—C10—C11—C12	−178.8 (3)
C2—C3—C4—O2	−179.4 (3)	C10—C11—C12—C13	0.0 (6)
O1—C3—C4—C5	−175.8 (3)	C11—C12—C13—F1	−180.0 (3)
C2—C3—C4—C5	3.3 (5)	C11—C12—C13—C14	1.0 (6)
O2—C4—C5—C6	−177.4 (3)	F1—C13—C14—C15	179.3 (3)
C3—C4—C5—C6	−0.5 (5)	C12—C13—C14—C15	−1.7 (6)
C2—C1—C6—C5	1.6 (5)	F1—C13—C14—Br1	−2.8 (5)
C2—C1—C6—C7	−175.4 (3)	C12—C13—C14—Br1	176.2 (3)
C4—C5—C6—C1	−2.0 (5)	C13—C14—C15—C10	1.2 (5)
C4—C5—C6—C7	175.1 (3)	Br1—C14—C15—C10	−176.6 (2)
C1—C6—C7—O3	174.9 (3)	C11—C10—C15—C14	−0.2 (5)
C5—C6—C7—O3	−2.1 (5)	C9—C10—C15—C14	178.2 (3)
C1—C6—C7—C8	−1.9 (5)		

### Hydrogen-bond geometry (Å, º)

**Table:**

*D*—H···*A*	*D*—H	H···*A*	*D*···*A*	*D*—H···*A*
C15—H15*A*···O2^i^	0.93	2.61	3.506 (5)	162
C11—H11*A*···O3^ii^	0.93	2.46	3.358 (5)	162

Symmetry codes: (i) −*x*+3/2, *y*−1/2, −*z*+1/2; (ii) −*x*+1, −*y*+2, −*z*.
